# Spatial correlation-based quadratic cost function for wavefront shaping through scattering media

**DOI:** 10.1117/1.JBO.29.11.115002

**Published:** 2024-11-20

**Authors:** Amit Kumar, Ayush Sharma, S. K. Biswas

**Affiliations:** Indian Institute of Science Education and Research Mohali, Department of Physical Sciences, Bio-NanoPhotonics Lab, Manauli, Punjab, India

**Keywords:** wavefront shaping, cost function, spatial light modulator, ℓ_2_ quadratic norm, quadratic cost function

## Abstract

**Significance:**

The feedback-based wavefront shaping emerges as a promising method for deep tissue microscopy, energy control in bio-incubation, and re-configurable structural illuminations. The cost function plays a crucial role in the feedback-based wavefront optimization for focusing light through scattering media. However, popularly used cost functions, such as intensity (η) and peak-to-background ratio (PBR) struggle to achieve precise intensity control and uniformity across the focus spot.

**Aim:**

We have proposed an $\ell_{2}$-norm-based quadratic cost function (QCF) for establishing both intensity and position correlations between image pixels, which helps to advance the focusing light through scattering media, such as biological tissue and ground glass diffusers.

**Approach:**

The proposed cost function has been integrated into the genetic algorithm, establishing pixel-to-pixel correlations that enable precise and controlled contrast optimization, while maintaining uniformity across the focus spot and effectively suppressing the background intensity.

**Results:**

We have conducted both simulations and experiments using the proposed QCF, comparing its performance with the commonly used η and PBR-based cost functions. The results evidently indicate that the QCF achieves superior performance in terms of precise intensity control, uniformity, and background intensity suppression. By contrast, both the η and PBR cost functions exhibit uncontrolled intensity gain compared with the proposed QCF.

**Conclusions:**

The proposed QCF is most suitable for applications requiring precise intensity control at the focus spot, better uniformity, and effective background intensity suppression. This method holds significant promise for applications where intensity control is critical, such as photolithography, photothermal treatments, dosimetry, and energy modulation within and outside bio-incubation systems.

## 1 Introduction

Biological tissue is a highly scattering medium, where light propagation encounters repeated random scattering, leading to unavoidable wavefront distortion and the generation of speckle noise.^1^ This wavefront distortion poses a significant challenge to focusing light and forming the desired light pattern through or within the scattering media.^1^ Wavefront shaping and light pattern formation through highly scattering materials hold significant potential across various fields. These include targeting deep neurons with structured light for optogenetics,^2^ deep tissue fluorescence imaging,^3^ and structured illumination light microscopy.^4^ In addition, generating patterns through turbid media is advantageous for material processing and energy delivery in medical sciences.^5^ There is also a growing demand for complex structure formation using wavefront shaping through scattering media for applications such as 3D holographic displays.^6^^–^^8^

Nevertheless, speckle noise generated in the scattering process can be mitigated by modulating the incident wavefront. By employing an integrative feedback-based wavefront shaping technique with an appropriate cost function, it is possible to generate the desired light patterns through or within scattering media.^5^^,^^9^^–^^24^ The experimental demonstration of focusing light through scattering media using a spatial light modulator (SLM) was first achieved by Vellekoop and Mosk.^11^ Various methods have been employed to focus light through scattering media, including iterative optimization, transmission matrix, and digital optical phase conjugation approaches.^5^^,^^9^^–^^11^^,^^15^^–^^25^ Recent research indicates that meta-heuristic optimization algorithms, such as genetic algorithms (GAs), are particularly effective for wavefront manipulation in scattering media, offering superior enhancement performance even in conditions with high noise levels.^19^^–^^24^

In standard iterative methods, the optimizing cost function serves as the primary guide for reaching the desired solution.^19^^–^^24^ Within the realm of wavefront shaping, several notable cost functions have been identified, including target intensity (η),^11^ peak-to-background ratio (PBR),^22^ and standard deviation.^26^ Vellekoop and A.P. Mosk first utilized an intensity-based cost function (η) for feedback-based wavefront optimization in 2007,^11^ defining it as the ratio of the optimized intensity at the target spot to the average intensity “before optimization.” This target intensity-based cost function (η) provided feedback solely on the target location while neglecting the background feedback. To enhance background suppression, Zhang et al.^22^ introduced a PBR-based cost function in 2017, defined as the ratio of the optimized intensity at the target spot to the “optimized intensity” in the background. Both intensity and PBR cost functions are effective for focus-spot formation. However, focusing light of a specific set value of intensity in a controlled manner, with sufficient uniformity at the region of interest, poses a significantly greater challenge compared with ordinary spot focusing,^24^^,^^26^^,^^27^ where a set value is a particular intensity grayscale value of an 8-bit camera image consisting 256 intensity grayscale levels. When attempting to form a precise intensity target using conventional η and PBR cost functions, the target’s contrast is enhanced randomly with uncontrolled intensity gain over the target pixels. Both the abovementioned η and PBR cost functions completely lack the pixel-to-pixel intensity correlation and position correlation of the target pixels.

In 2012, Conkey et al.^26^ introduced a cost function based on standard deviation and total intensity to implement in the GA aimed at focusing multiple spots. The cost function is calculated by summing all the intensities of the focus spots ($\sum_{m=1}^{M}I_{m}$) and subtracting the product of the number of focus spots (M) and the standard deviation of their intensities (σ(I)), i.e., $\text{Cost function}=\overset{M}{\underset{m=1}{\sum }}I_{m}-M/\sigma (I)$.^26^ This approach primarily enhances intensity and distribution across multiple focus spots, however, does not account for the background noise suppression or continuous structure formation.^26^

In 2019, Feng et al.^28^ introduced a hybrid optimization scheme based on the non-dominated sorting GA II (NSGA2-H) for multi-point focusing. This approach utilized two objectives: the first being the total intensity of “M” focus points divided by the average intensity of the output light field, and the second objective being the standard deviation divided by the average of the focus points’ intensity.^28^

It is quite challenging to achieve the intensity as per a set value in a controlled manner at the focus-spot area, as it requires a cost function based on both intensity and position correlations to optimize intensity distribution at the target plane. In this article, the performance of traditionally used cost functions has been evaluated within the iterative GA, leading to the design of an $\ell_{2}$-norm-based quadratic cost function (QCF) to enhance precise control of contrast at the focus-spot location while maintaining the uniformity of intensity distribution at the focus-spot area. Both experiments and simulations have been conducted to demonstrate the performance of the commonly used η and PBR cost functions along with the proposed QCF. The following sections present the formulation of the proposed $\ell_{2}$-norm-based QCF, experimental results, simulation results, discussion, and conclusions.

## 2 Methods

Both intensity and position correlations between the reference image ($I_{\mathrm{ref}}$) and the reconstructed (obtained) image ($I_{O}$) have been established using the proposed $\ell_{2}$-norm-based QCF, where the optimization process considers pixel-to-pixel level correlations in terms of both intensity and position.

The $\ell_{2}$-norm,^29^ often known as Euclidean norm, is a mathematical and computational method to calculate the length or magnitude of a vector in a multidimensional space. The $\ell_{2}$-norm is popularly used in mathematics,^29^ physics,^30^ machine learning,^31^ and convex optimization.^32^ If I is an element of n-dimensional vector space with basis $e_{i}$, then I can be written as $I=\sum_{i=1}^{n}U_{i}e_{i}$, where $U_{i}$ is the component of I along the basis $e_{i}$. Therefore, an $\ell_{2}$-norm-based QCF is formulated with the summation of the $\ell_{2}$-norm of the background pixel intensity of the reconstructed image ($I_{O}^{B}$) and reference image ($I_{\mathrm{ref}}^{B}$), and the square of $\ell_{2}$-norm of focus-spot pixel intensity of the reconstructed image ($I_{O}^{T}$) and reference image ($I_{\mathrm{ref}}^{T}$), multiplied by a constant weight, ξ. The formulated QCF can be written as follows:

$$\text{Quadratic cost function}(\mathrm{QCF})=-[\sqrt{\overset{m}{\underset{i=1}{\sum }}|I_{O,i}^{B}-I_{\mathrm{ref},i}^{B}{|}^{2}}+\xi (\overset{n}{\underset{j=1}{\sum }}|I_{O,j}^{T}-I_{\mathrm{ref},j}^{T}{|}^{2})].$$ (1)

In the above equation, the self-determined constant weight factor ξ is considered for balancing the magnitude between the background and the focus-spot terms of QCF. This constant is defined using the initial intensity distribution of the background and the focus spot formed in the camera image by displaying the first phase mask of the first generation on the SLM only. The equation for estimating the ξ is expressed as follows:

$$\xi =\frac{{\left(\sqrt{\overset{m}{\underset{i=1}{\sum }}|I_{O,i}^{B}-I_{\mathrm{ref},i}^{B}{|}^{2}}\right)}_{\mathrm{Gen}=1}}{{\left(\overset{n}{\underset{j=k}{\sum }}|I_{O,j}^{T}-I_{\mathrm{ref},j}^{T}{|}^{2}\right)}_{\mathrm{Gen}=1}}.$$ (2)

In the abovementioned equations [Eqs. (1) and (2)], i and j denote the indices corresponding to background and focus-spot pixels, respectively. The notation m represents the total number of background pixels, and n signifies the total number of focus-spot pixels. The developed QCF is proficient in both background optimization and intensity gain at the focus-spot position. The first term [Eq. (1)] of the QCF aims to minimize background intensity, whereas the second quadratic term tries to gain the intensity at the focus spot in a controlled manner as per the pre-decided set value. In subsequent sections detailing experimental and simulation outcomes, it has been observed that the proposed QCF controls the intensity at the focus-spot area as per the required set value of intensity, thereby avoiding overshooting of the desired intensity levels.

### 2.1 Experimental Setup

**Fig. 1:**
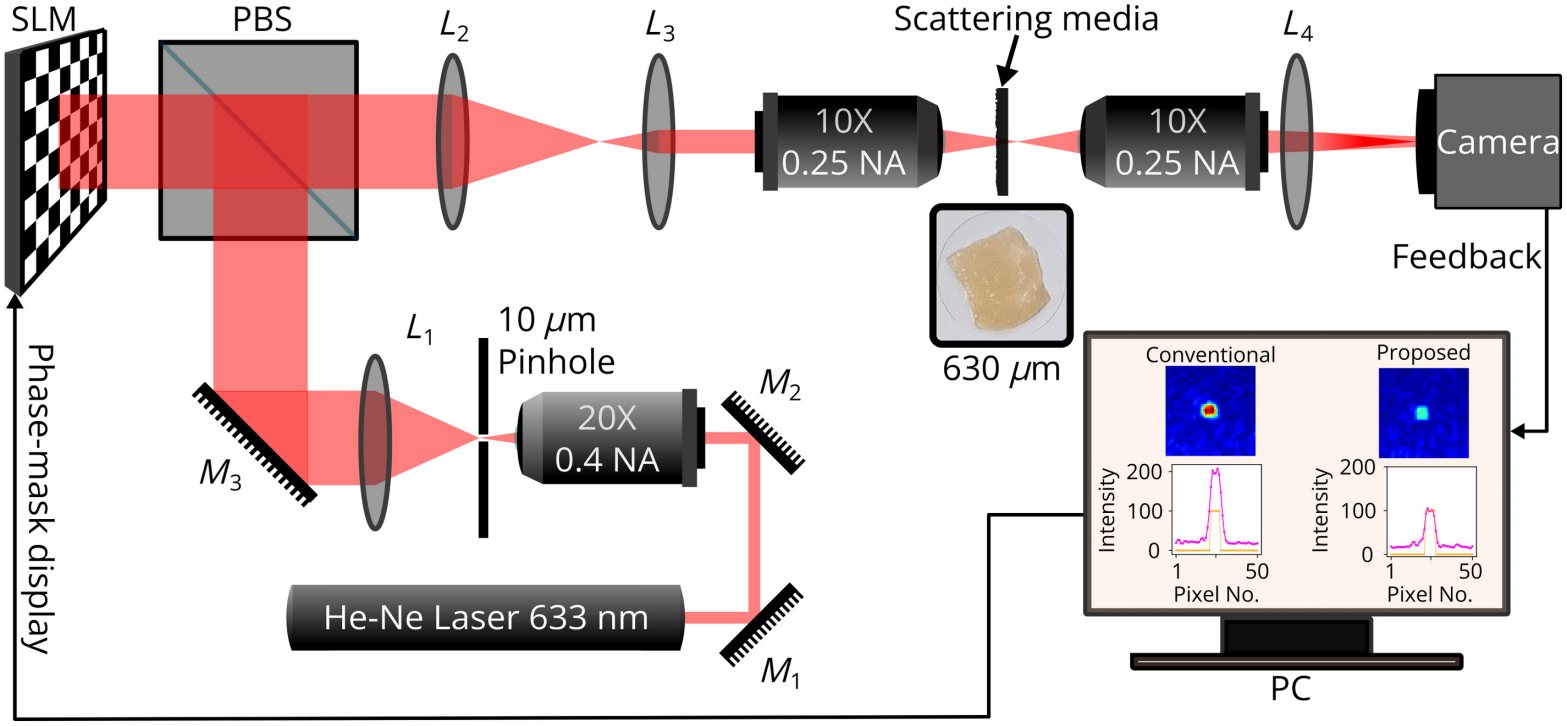
Schematic representation of the experimental setup with binary phase SLM. Coherent light from the He-Ne laser is incident on PBS. The reflected light from the PBS is directed onto the SLM. This modulated wavefront is made to pass through a 10× objective by a 4F system comprising lenses $L_{2}$ and $L_{3}$. The scattering media is kept at the Fourier plane of the 10× objective. The wavefront from this plane is imaged by a camera after passing through a 10× collecting objective and lens $L_{4}$. The camera provides the necessary feedback to calculate the cost and update the phase mask.

The experimental setup consists of a binary ferroelectric liquid crystal-based spatial light modulator (FLC-SLM) with 1280×1024  pixels for modulating the incoming wavefront by introducing a spatial phase delay of either 0 or π. A comprehensive schematic of the experimental setup is presented in Fig. 1. A 12 mW He-Ne laser (633 nm, Newport Corporation, Irvine, United States) delivering vertically linearly polarized light with a polarization ratio of 500:1 is used in the system. Along the path, a spatial filter system (Thorlabs, KT311/M, Newton, United States), comprising a pinhole (ϕ=10  μm) and an objective (20×, Numerical Aperture = 0.40), is employed to eradicate higher-order noise from the beam. After that, the spatially filtered diverging beam is collimated using a lens $L_{1}$ (f=250  mm) to achieve a flat beam profile on the FLC-SLM surface. The wavefront modulated by the FLC-SLM passes through a 4F setup and enters an objective (10×, NA=0.25), which focuses the wavefront onto the surface of the scattering medium. Before entering the scattering medium, the power of the incident beam is measured at 0.74 mW. A secondary objective (10×, NA=0.25) is situated behind the scattering medium to collect the reconstructed light. The light collected by the second objective is sent toward an 8-bit CMOS camera (Thorlabs, DCC3260C) for imaging and feedback purposes.

The modulated binary phase masks are sequentially displayed on the SLM for wavefront modulation. This modulated wavefront is propagated toward the ground glass (GG) diffuser of 220-grit size (Edmund Optics, Barrington, United States) or the biological tissue sample. A series of lenses positioned along the modulated wavefront path facilitates the acquisition of image plane data by a camera, furnishing the necessary feedback signal for optimizing the phase mask. Triggered by an SLM-interfaced pulse generator, the camera captures images utilized to estimate the cost function for the particular phase mask. Figure 1 shows the schematic of the experimental setup. A photograph of the experimental setup is given in Fig. S10 in the Supplementary Material. A widely adapted GA has been considered^19^^–^^24^ in the optimization model. Different cost functions are used to scrutinize and validate their performance in the optimization process.

### 2.2 Simulation Details

The simulation model is designed in the Python 3 programming language. The computing system consists of Intel Xeon E5-2690 V4 2.60 GHz × 28 CPU, 128 GB RAM, and Ubuntu 22.04.3 operating system. Input modes (M) span a domain of 40×40  pixels, whereas output modes (N) occupy a 25×25  pixel domain. The transmission matrix (T) of dimensions M×N is generated using a complex Gaussian random distribution ($\mu_{T}=0$ and $\sigma_{T}=0.05$) to mimic scattering.^19^^,^^27^ A 30% noise (δ) is introduced to output mode intensity to mimic the experimental conditions. A widely adapted GA has been considered^19^^–^^24^ to model the optimization for a viable solution. Initially, a population (P=100) of random binary phase masks is generated using a discrete uniform distribution of values 0 and 255, corresponding to the 0 and π phases, respectively. The phase masks are ranked according to their fitness value obtained in output mode, guiding parent selection and subsequent iterations. A crossover rate ($r_{c}$) of 50% is maintained, whereas the initial mutation rate starts at 1%, decaying exponentially with a constant rate (λ).^19^^,^^27^^,^^33^

## 3 Results

### 3.1 Experimental Results

**Fig. 2:**
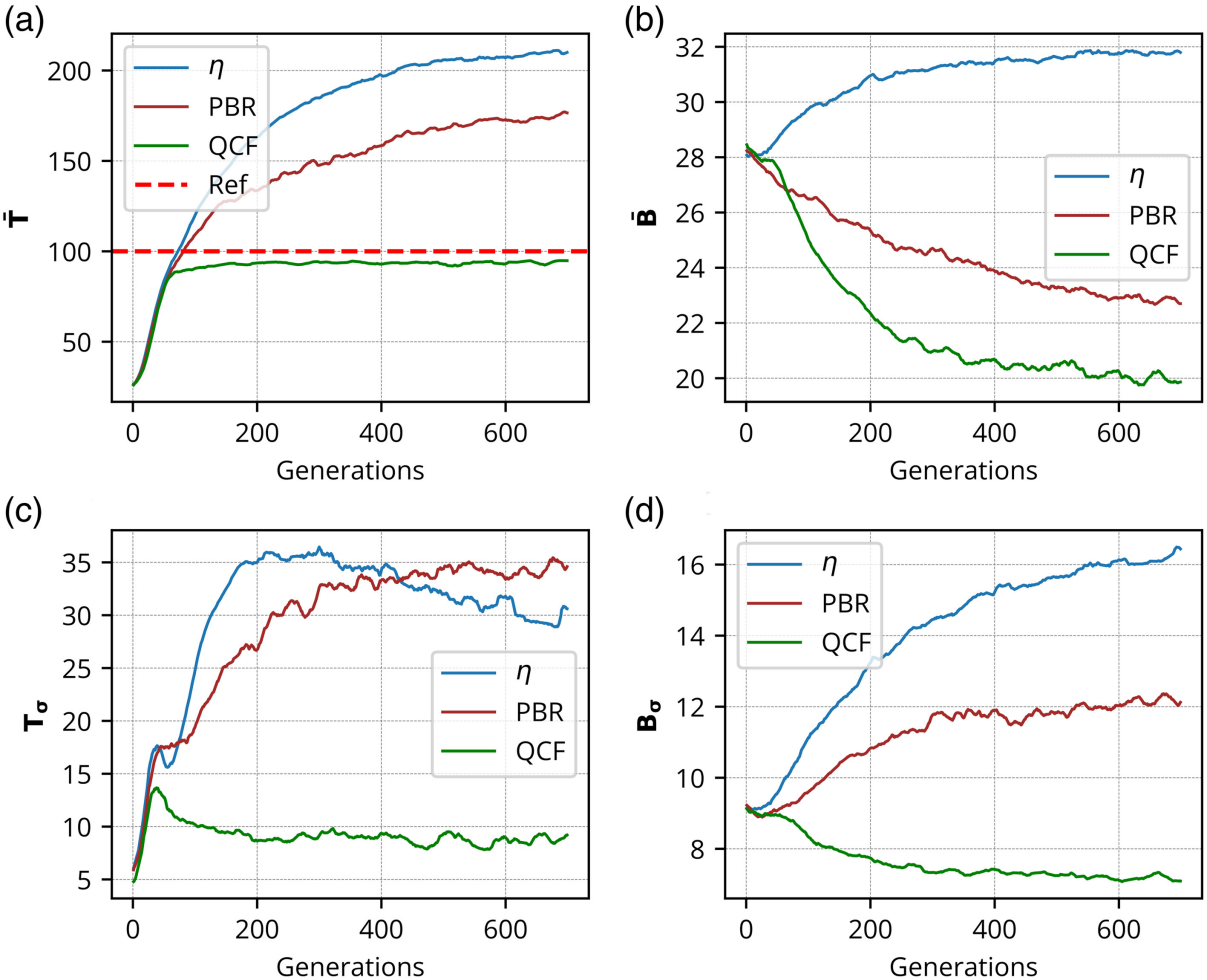
Experimental results for the formation of the focus spot consisting of 100 grayscale through GG diffuser: This shows intensity analysis for the focus spot consisting of 100 grayscale where panel (a) shows the mean intensity of the focus spot ($\overset{¯}{T}$), panel (b) shows background intensity ($\overset{¯}{B}$), panel (c) shows the standard deviation of focus-spot intensity ($T_{\sigma }$), and panel (d) shows the standard deviation of background intensity ($B_{\sigma }$), over generations, for η, PBR, and QCF.

**Fig. 3:**
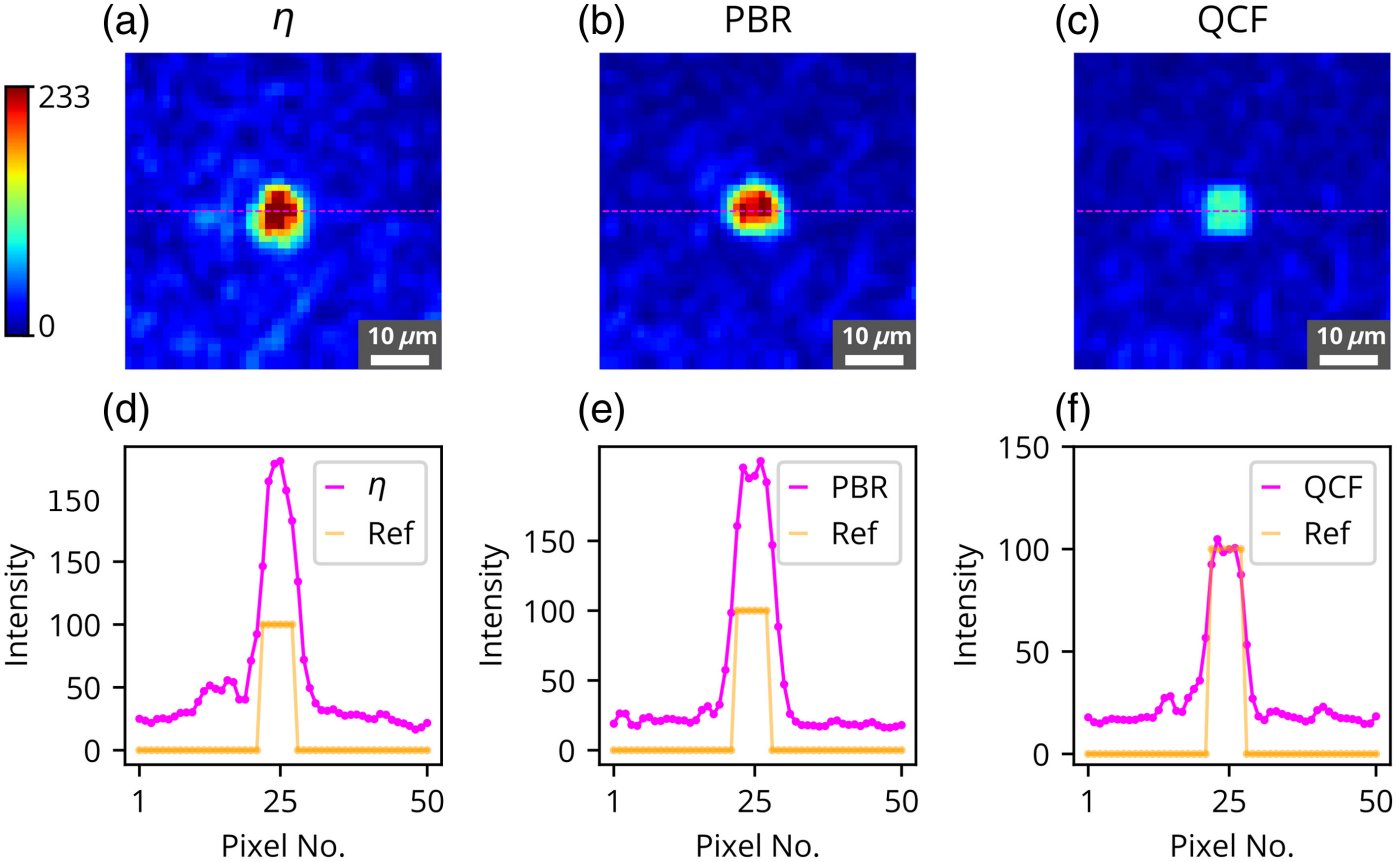
Experimental results for the formation of the focus spot consisting of 100 grayscale through GG diffuser where reconstructed images are shown for the focus spot consisting of 100 grayscale using (a) η, (b) PBR, and (c) QCF. Horizontal cross-section intensity lineplot comparison for the reference image and the reconstructed image is shown for the cost functions (d) η, (e) PBR, and (f) QCF.

**Fig. 4:**
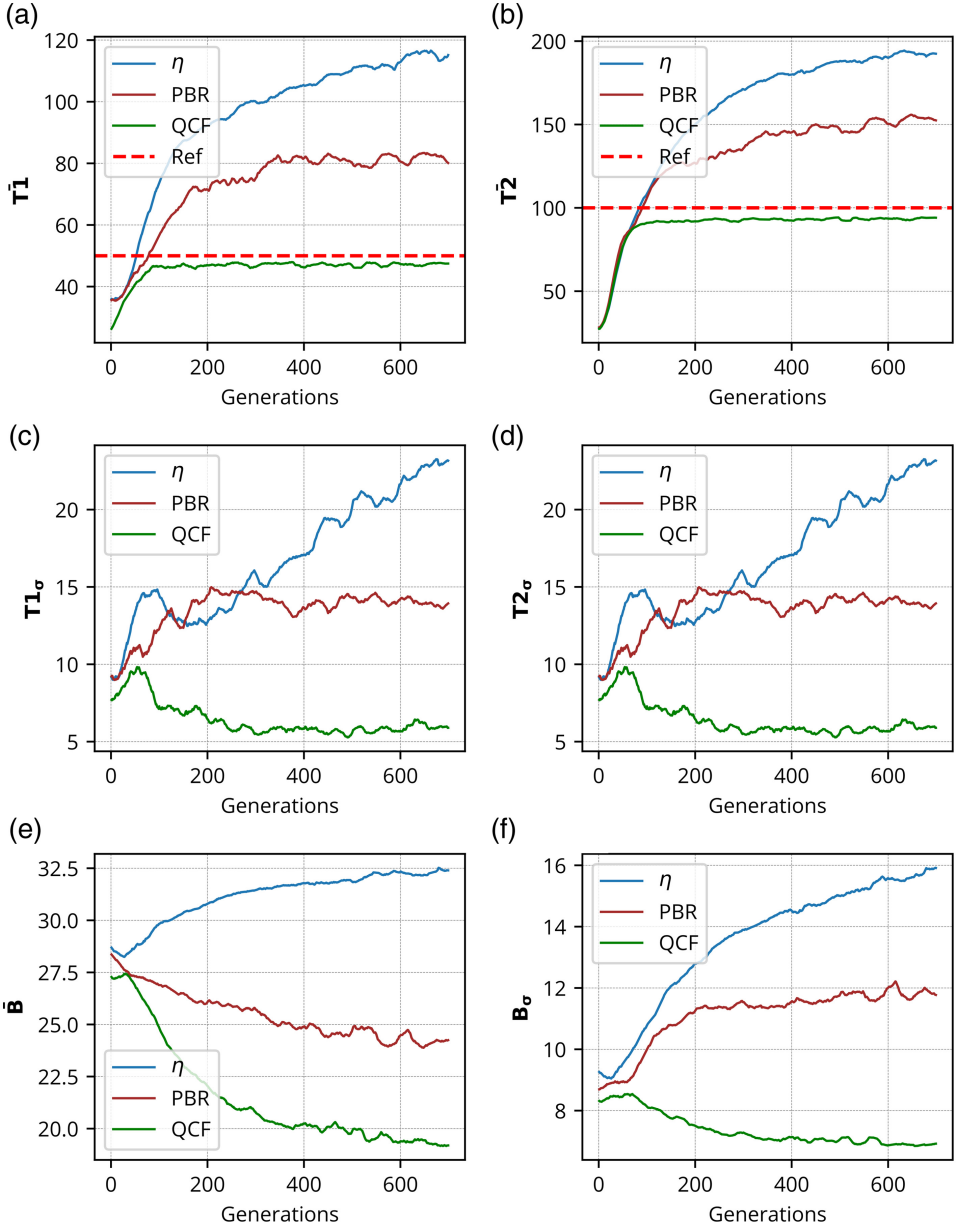
Experimental results for the formation of the dual focus spots consisting of 50 and 100 grayscale through GG diffuser: Intensity analysis for dual focus spots, the first focus spot consisting of 50 grayscale and the second consisting of 100, where panel (a) shows the mean intensity of the first focus spot ($\overset{¯}{T}1$), panel (b) shows the mean focused intensity of second spot ($\overset{¯}{T}2$), panel (c) shows the intensity standard deviation of the first focus spot ($T1_{\sigma }$), panel (d) shows the intensity standard deviation of the second focus spot ($T2_{\sigma }$), panel (e) shows the mean background intensity ($\overset{¯}{B}$), and panel (f) shows the standard deviation of background intensity ($B_{\sigma }$), over generations, for η, PBR, and QCF.

**Fig. 5:**
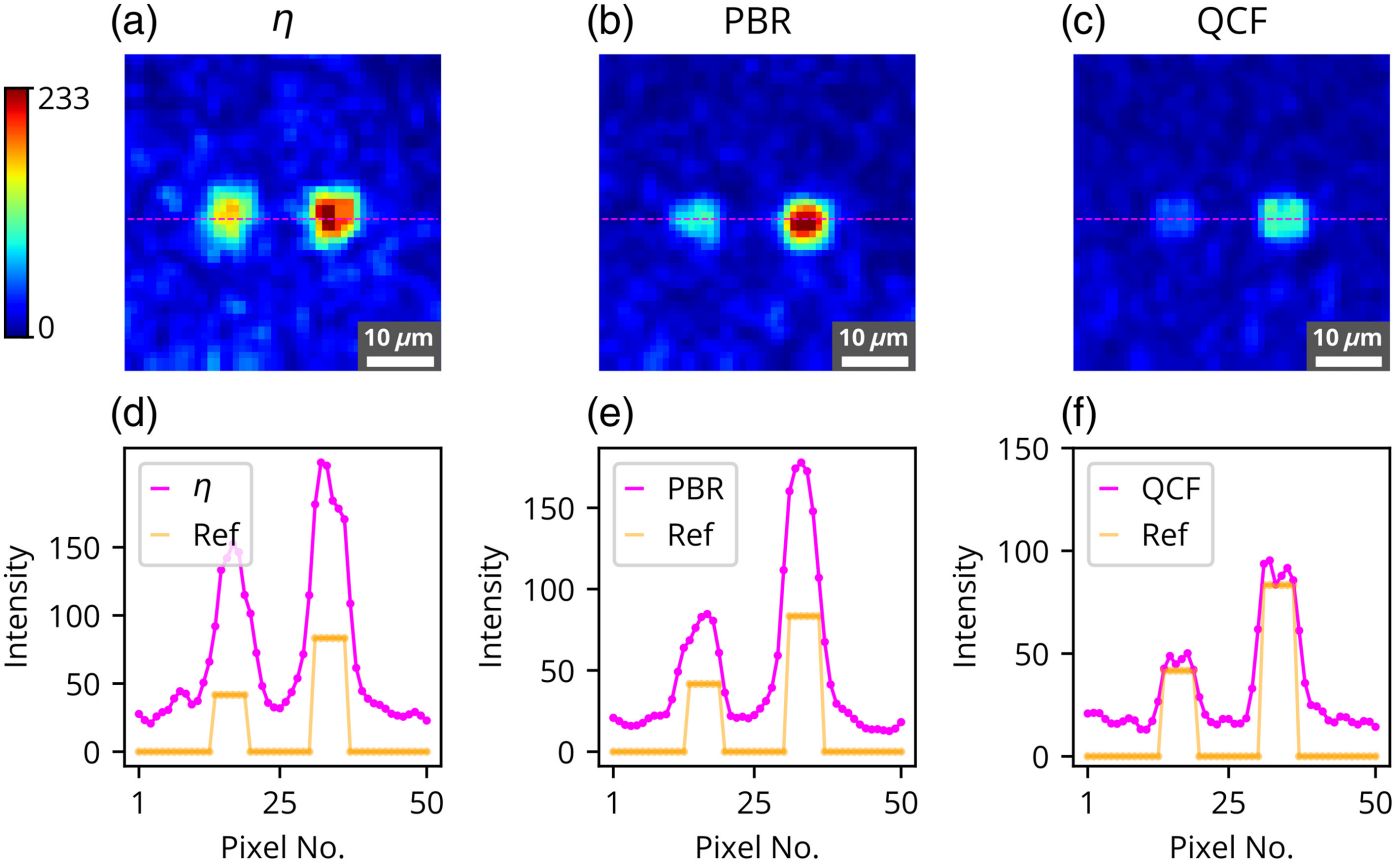
Experimental results for the formation of dual focus spots consisting of 50 and 100 grayscale through GG diffuser where final images are shown for dual focus spots, the first focus spot consisting of 50 grayscale, and the second focus spot consisting of 100 grayscale using (a) η, (b) PBR, and (c) QCF. The horizontal cross-section intensity lineplot comparison for the reference image and the reconstructed image is also shown for (d) η, (e) PBR, and (f) QCF.

**Fig. 6:**
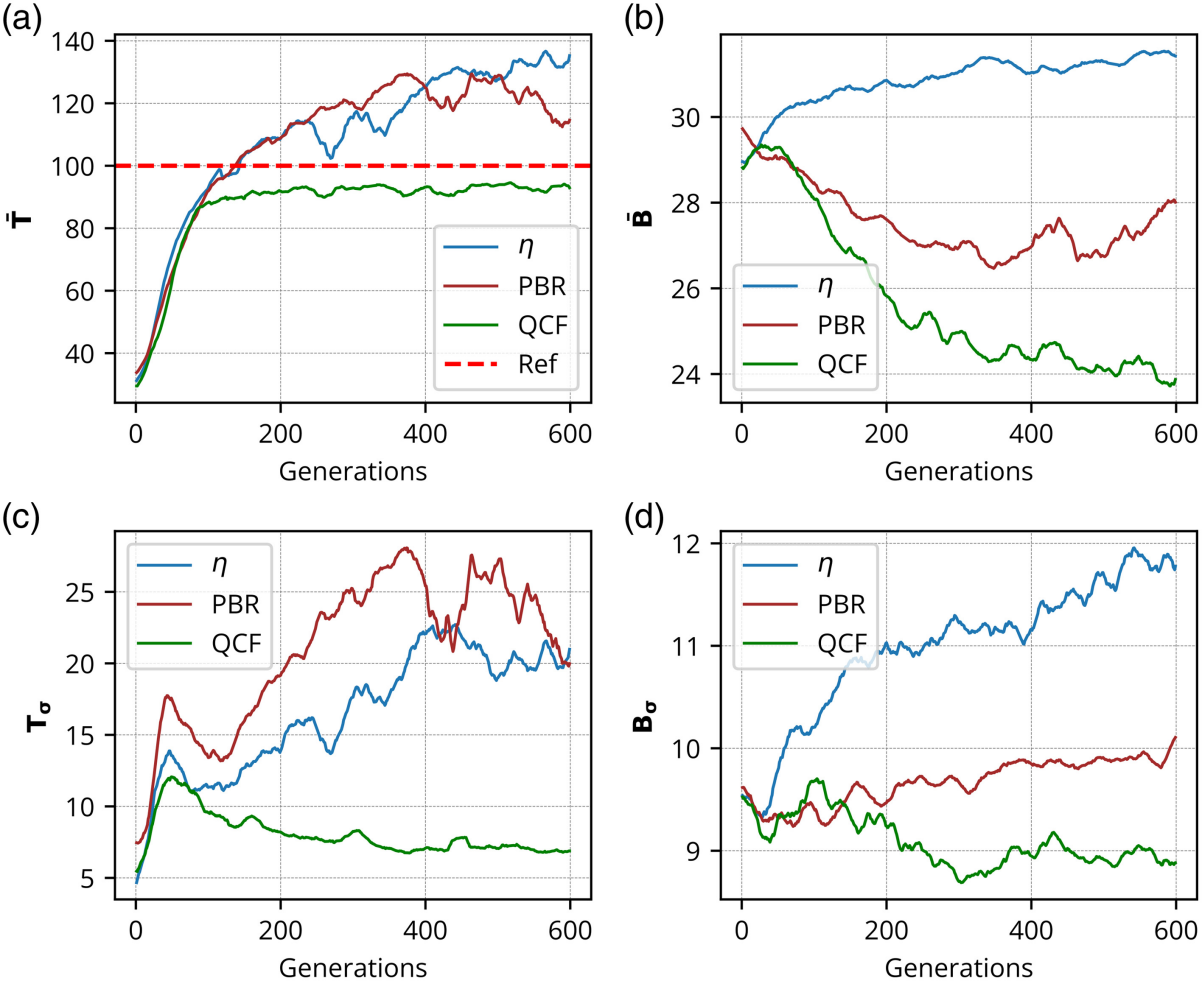
Experimental results for the formation of the focus spot consisting of 100 grayscale through chicken tissue of 630  μm thickness: This shows the intensity analysis for the focus spot consisting of 100 grayscale where panel (a) shows the mean intensity of the focus spot ($\overset{¯}{T}$), panel (b) shows the background intensity ($\overset{¯}{B}$), panel (c) shows standard deviation of focus spot intensity ($T_{\sigma }$), and panel (d) shows the standard deviation of background intensity ($B_{\sigma }$), over generations.

**Fig. 7:**
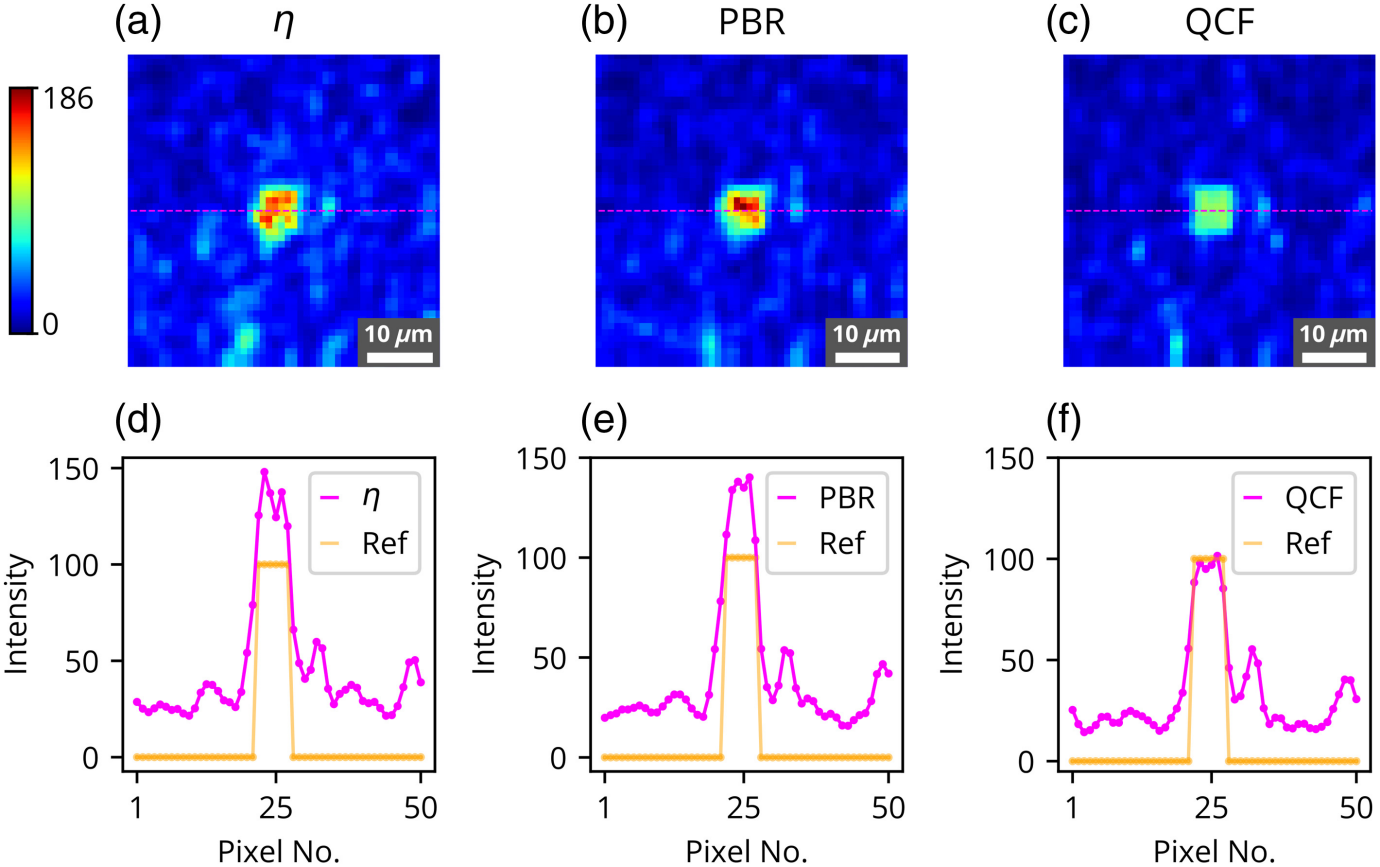
Experimental results for the formation of the focus spot consisting of 100 grayscale through chicken tissue of 630  μm thickness, where reconstructed images are shown for the focus spot consisting of 100 grayscale using (a) η, (b) PBR, and (c) QCF. Horizontal cross-section intensity lineplot comparison for the reference image and the reconstructed image is also shown for (d) η, (e) PBR, and (f) QCF.

**Fig. 8:**
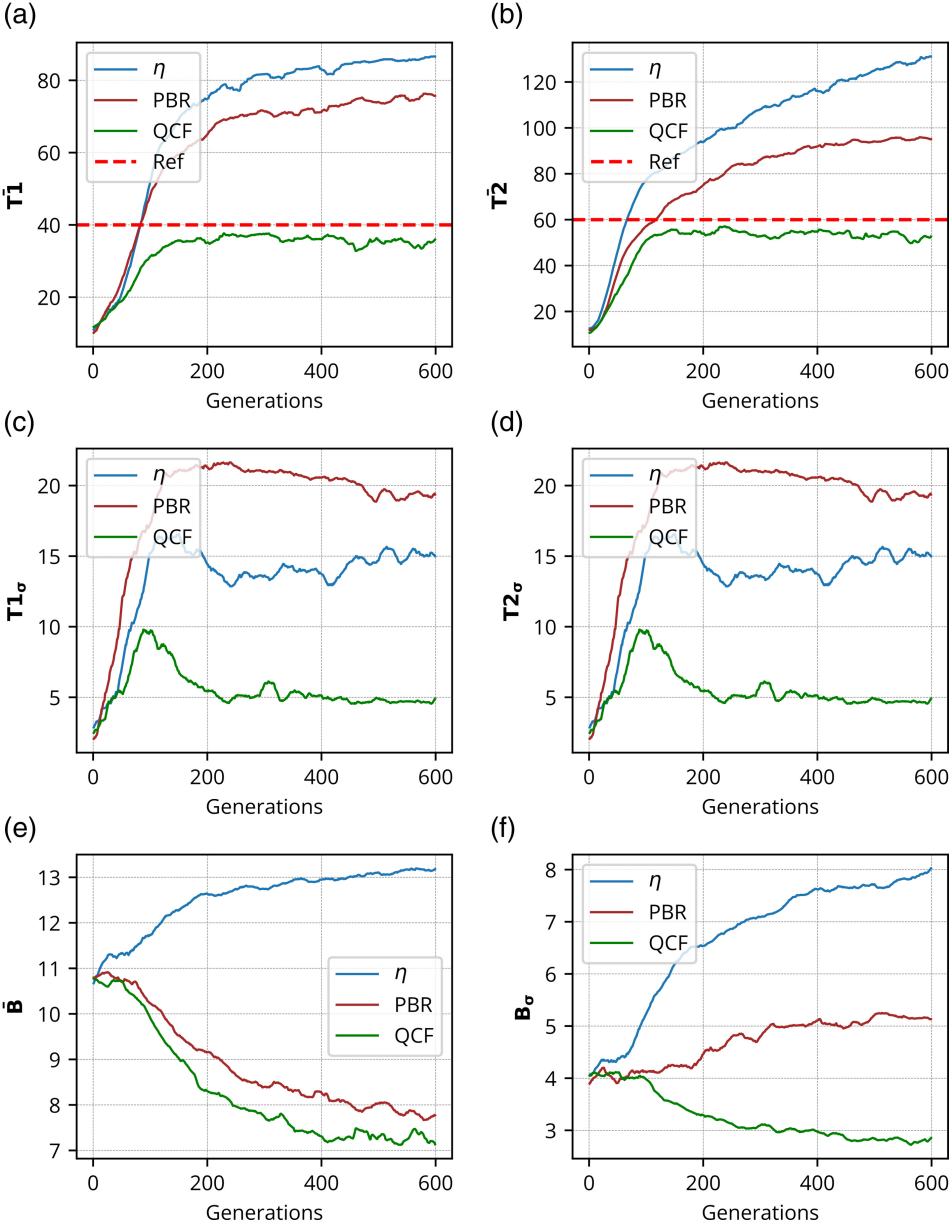
Experimental results for the formation of dual focus spots consisting of 40 and 60 grayscale through chicken tissue of 630  μm thickness: Intensity analysis for dual focus spots, the first focus spot consisting of 40 grayscale, and the second focus spot consisting of 60 grayscale where panel (a) shows the mean intensity of the first focus spot ($\overset{¯}{T}1$), panel (b) shows the mean intensity of the second focus spot ($\overset{¯}{T}2$), panel (c) shows the standard deviation of the first focus spot intensity ($T1_{\sigma }$), panel (d) shows the standard deviation of the second focus spot intensity ($T2_{\sigma }$), panel (e) shows the mean background intensity ($\overset{¯}{B}$), and panel (f) shows the standard deviation of background intensity ($B_{\sigma }$), over generations.

**Fig. 9:**
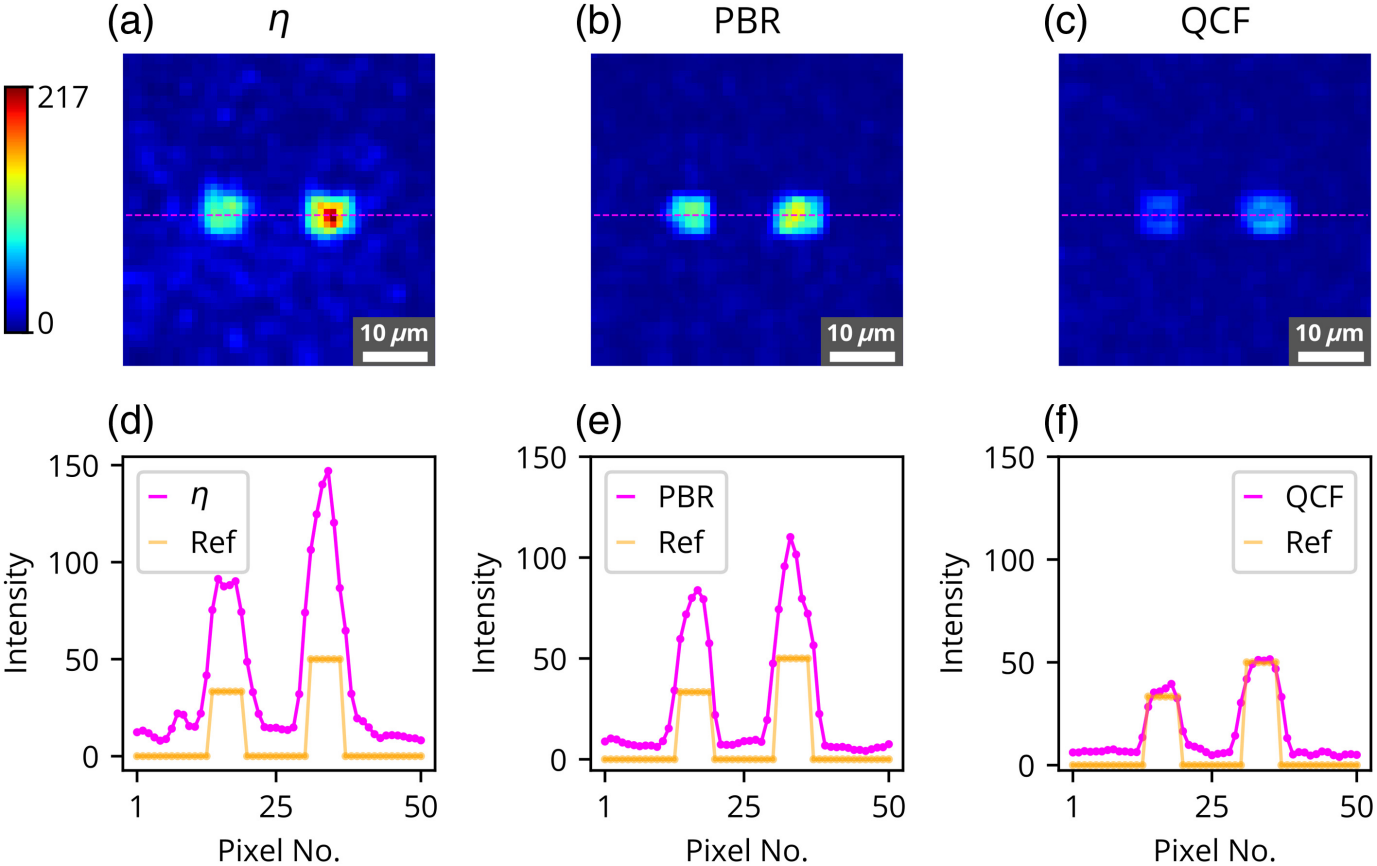
Experimental results for the formation of dual focus spots consisting of 40 and 60 grayscale through the chicken tissue of 630  μm thickness, where reconstructed images are shown for dual focus spots, the first focus spot consisting of 40 grayscale, and the second focus spot consisting of 60 grayscale using (a) η, (b) PBR, and (c) QCF. Horizontal cross-section intensity lineplot comparison for the reference image and the reconstructed image is also shown for (d) η, (e) PBR, and (f) QCF.

The η,^11^ PBR,^22^ and the proposed $\ell_{2}$-norm-based QCF have been rigorously investigated in the experimental framework utilizing a 220-grit GG diffuser and a chicken tissue of 630  μm thickness. These experimental outcomes are shown in Figs. 2–9. Figure 2 presents the experimental results of the intensity analysis for a single focus spot aimed at achieving 100 grayscale units. Figure 2(a) shows the mean intensity at the focus spot ($\overset{¯}{T}$) while optimizing the photon distribution at the Fourier plane using the cost functions η, PBR, and proposed QCF. Figure 2(b) shows background intensity variation ($\overset{¯}{B}$) with each generation, Fig. 2(c) shows the standard deviation of the focus-spot intensity ($T_{\sigma }$), and Fig. 2(d) shows the standard deviation of background intensity ($B_{\sigma }$), across generations. It is evident that the QCF demonstrates precise achievement of the focus-spot intensity of 100 grayscale units at the focus spot. Conversely, both η and PBR overshoot the intensity limit and fail to achieve uniform intensity distribution across the focus-spot area [Fig. 2(a)]. The proposed QCF is equally able to provide superior background noise suppression and controlled intensity enhancement at the focus spot as per the predecided set value [Fig. 2(b)]. Furthermore, the QCF achieves better (lower) standard deviation at both the focus-spot area and the background area compared with the η and PBR cost functions [Figs. 2(c) and 2(d)]. Figures 3(a)–3(c) show the final reconstructed focus-spot images with η, PBR, and proposed QCF, respectively. Figures 3(a)–3(c) evidently demonstrate that neither η nor PBR succeeds in achieving the desired intensity at the focus spot within the specified limit. Figures 3(d)–3(f) depict the horizontal cross-section lineplot for the required (yellow color) and reconstructed (magenta color) focus-spot intensity. The lineplot profiles demonstrate that both η and PBR overshoot the intensity limit and fail to achieve uniform intensity distribution over the focus-spot area. The reference images of different grayscale focus spots are shown in Fig. S1 in the Supplementary Material, and complete additional analyses for the formation of focus spots with 50 and 150 grayscale units through the GG diffuser are presented in Figs. S2–S5 in the Supplementary Material. We have scanned the entire grayscale range starting from 10 to 255 grayscale for the focus spot, and the results are discussed in Supplementary Material (Sec. 2.2, Fig. S6 in the Supplementary Material).

Similarly, the performance of the proposed cost function has been evaluated using dual focus spots, where two-level contrast or intensity limits are set. Figure 4 shows the intensity analysis of two focus-spot formation, with the first focus-spot area set at 50 grayscale and the second one at 100 grayscale. Figures 4 and 5 show that the QCF effectively regulates intensity levels at both focus spots without exceeding the specified limits. Conversely, conventional methods such as the η and PBR cost functions exhibit intensity overshooting beyond the prescribed limits for both focus spots.

Figures 5(a)–5(c) depict the reconstructed images corresponding to two focus spots with different contrasts, and Figs. 5(d)–5(f) show the horizontal cross-section lineplot for the required (yellow color) and reconstructed (magenta color) intensity for dual focus spots. These cross-section lineplots illustrate that the proposed cost function QCF effectively attains the predetermined contrast levels in a controlled manner. Conversely, η and PBR cost functions demonstrate a tendency to surpass the intensity limit for both focus spots.

To assess the efficacy of the proposed QCF, its performance has been further analyzed using a biological specimen, specifically chicken tissue with a thickness of 630  μm. The chicken tissue has been implemented in the experimental setup, and the experiments have been conducted for the cost functions η, PBR, and QCF (refer to Fig. S9 in the Supplementary Material for a photograph of the chicken tissue). Figure 6 represents the mean intensity analysis [Figs. 6(a) and 6(b)] and standard deviation analysis [Figs. 6(c) and 6(d)] for a single focus spot consisting of 100 grayscale values. Figure 6 demonstrates that the developed QCF achieves superior mean background intensity ($\overset{¯}{B}$) suppression compared with η and PBR cost functions. By contrast, the η and PBR cost functions do not achieve an effective background intensity suppression. The QCF continuously performs background noise suppression, and the background noise suppression gets slowed down after 300 generations, and thereafter, it oscillates. However, the QCF surpasses both the η and PBR cost functions in background intensity suppression within just 200 generations. Figure 7 displays the reconstructed images for the single focus spot of 100 grayscale [Figs. 7(a)–7(c)] and the contrast lineplot analysis [Figs. 7(d)–7(f)]. Additional comprehensive analysis of single focus-spot formation consisting of 50 grayscale value through the chicken tissue is provided in Figs. S7 and S8 in the Supplementary Material.

Quantification of the reconstructed dual focus spots using the biological sample has been further performed, and the results are displayed in Figs. 8 and 9. Similar to the experiments involving the GG diffuser, two contrast levels (grayscale values of 40 and 60) have been evaluated with the dual focus spots in the experiments conducted with chicken tissue. Our observations indicate that the chicken tissue exhibits higher scattering and absorption properties compared with the 220-grit GG diffuser. Consequently, we selected grayscale levels of 40 and 60 for the construction of dual focus-spot formation with contrast variation through the chicken tissue.

Figure 8 shows the mean intensity analysis [Figs. 8(a), 8(b), and 8(e)], standard deviation analysis [Figs. 8(c), 8(d), and 8(f)], reconstructed dual focus-spot images [Figs. 9(a)–9(c)], and contrast lineplot analysis [Figs. 9(d)–9(f)] for different optimizing cost functions. Figure 9 shows the comparison of the set contrasts or intensity (in yellow color) and the achieved contrast level or intensity (in magenta color) at the dual focus spots. The contrast lineplots across the focus spots demonstrate that the proposed QCF achieves the set contrast levels in a precisely controlled manner. By contrast, both η and PBR overshoot the intensity limit for both the focus spots. These results evidently demonstrate the precise intensity and better (lower) uniformity achievement at the target focus spot with the proposed QCF compared with the commonly used η and PBR methods.

#### 3.1.1 Shape similarity test

The shape similarity analysis^34^ of the reconstructed focus spot has been conducted and the results are presented in this section. In this analysis, a reconstructed image ($I_{O}$) and a reference image ($I_{\mathrm{ref}}$) are compared using a specific similarity metric. This metric integrates cosine similarity and histogram parameters^34^ and is formulated as follows:

$$\text{Metric value}=[\frac{(\overset{¯}{T}-\overset{¯}{B})\times \overset{¯}{T}}{T_{\sigma }B_{\sigma }}]\times \text{cosine}(I_{O},I_{\mathrm{ref}}).$$ (3)

**Fig. 10:**
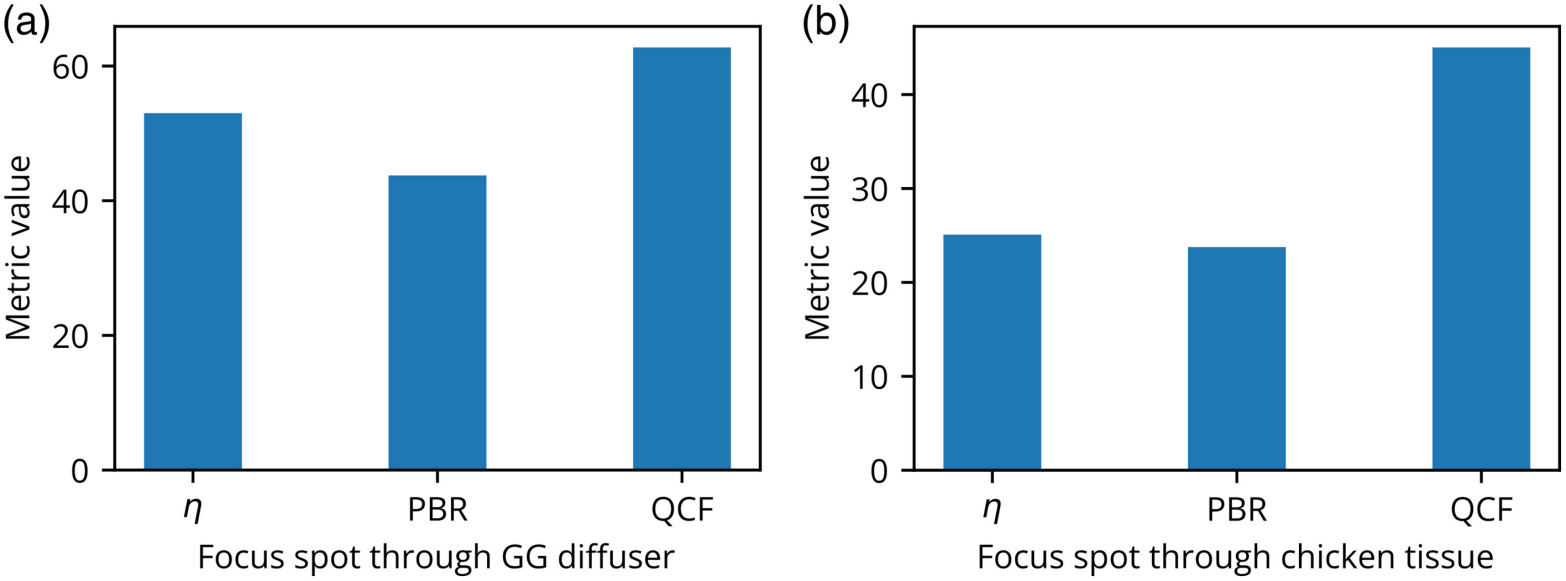
Metric value analysis for the single focus-spot formation in experiments: This shows the metric value analysis for the focus-spot formation of 100 grayscale through (a) 220-grit GG diffuser and (b) through the chicken tissue of 630  μm thickness. Both results show that QCF performs better in terms of metric value compared with η and PBR cost functions.

In the equation above [Eq. (3)], $\text{cosine}(I_{O},I_{\mathrm{ref}})$ represents the cosine similarity between the reconstructed image ($I_{O}$) and reference image ($I_{\mathrm{ref}}$). The parameters $\overset{¯}{T}$ and $\overset{¯}{B}$ denote the mean intensities of the focus spot and background pixels, respectively. The parameters $B_{\sigma }$ and $T_{\sigma }$ indicate the standard deviations of the background and focus-spot pixel intensities, respectively. Figure 10 presents the shape similarity analysis for the single focus spot consisting of 100 grayscale. Figure 10 clearly demonstrates that QCF achieves the highest metric value as compared with η and PBR for both the GG diffuser and chicken tissue sample cases.

### 3.2 Simulation Results

**Fig. 11:**
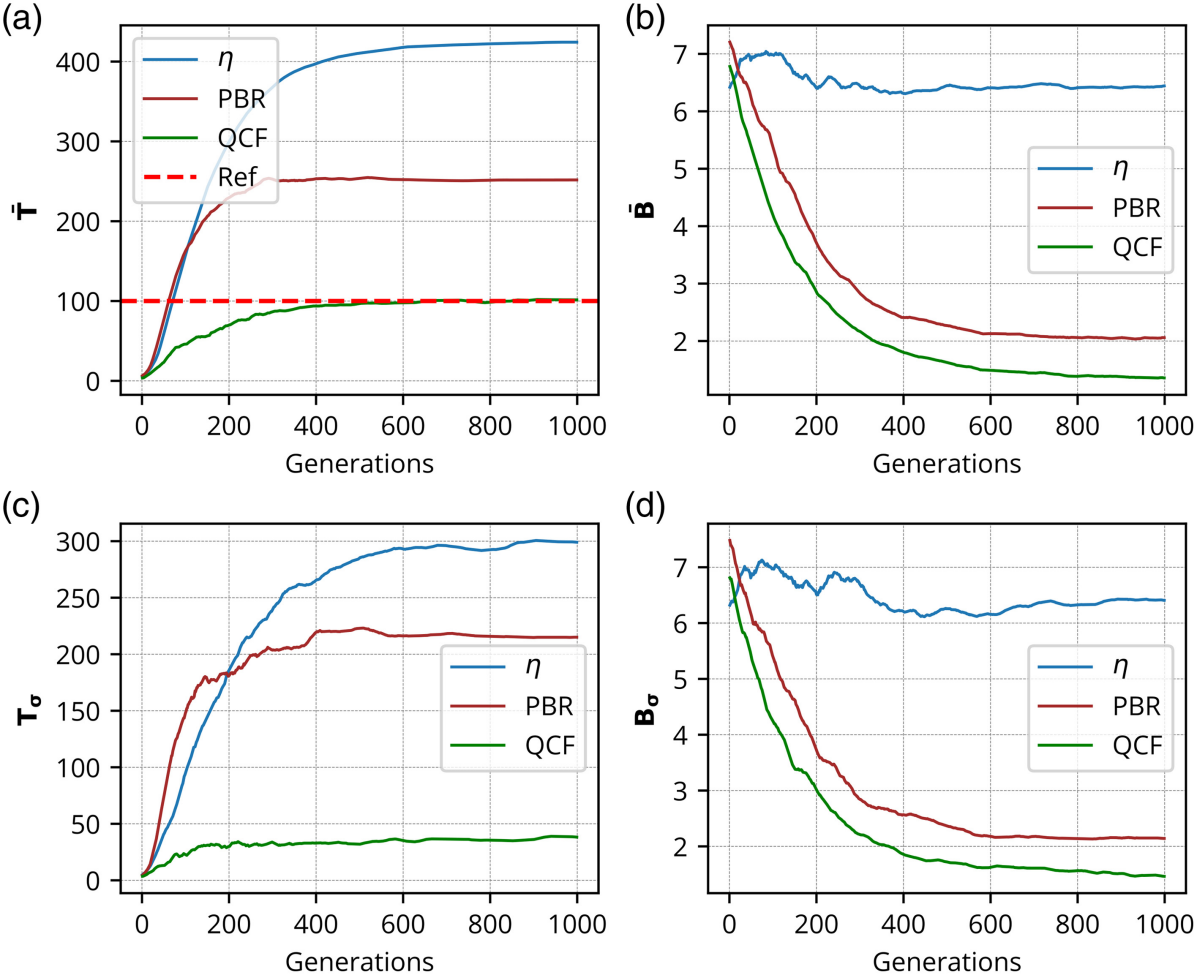
Simulation results: Intensity analysis for the focus spot consisting of 100 grayscale where panel (a) shows the mean intensity of the focus spot ($\overset{¯}{T}$), panel (b) shows the background intensity ($\overset{¯}{B}$), panel (c) shows the standard deviation of focus-spot intensity ($T_{\sigma }$), and panel (d) shows the standard deviation of background intensity ($B_{\sigma }$), over generations.

**Fig. 12:**
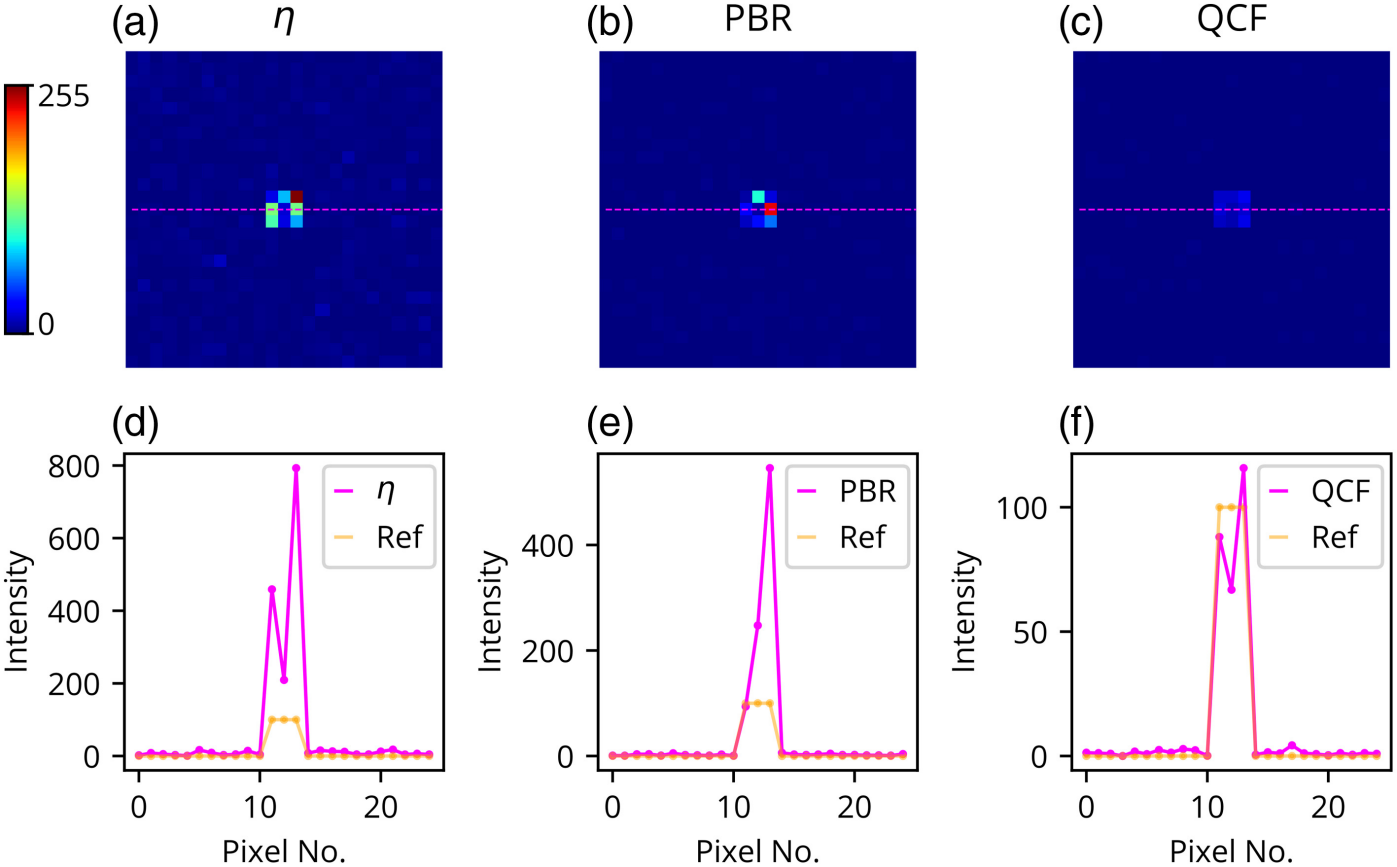
Simulation results, where reconstructed images are shown for the focus spot consisting of 100 grayscale using (a) η, (b) PBR, and (c) QCF. The intensity cross-section lineplot comparison for the reference image and the reconstructed image is also shown for (d) η, (e) PBR, and (f) QCF.

The QCF has been tested alongwith η and PBR cost functions in the widely adopted simulation model.^19^^–^^24^ Detailed descriptions of the simulation model are provided in the Methods section (Sec. 2.2). Figures 11 and 12 present the simulation results and demonstrate the ability to achieve precise intensity at the focus spot, whereas η and PBR overshoot the predecided intensity level and fail to maintain uniformity at the focus spot [Fig. 11(a)]. In addition, QCF exhibits superior background noise suppression compared with the η and PBR cost functions [Fig. 11(b)]. The QCF also achieves better (lower) standard deviation at both the focus-spot location and the background area, relative to the η and PBR cost functions [Figs. 11(c)–11(d)]. Contrast lineplots across the focus spot (Fig. 12) show that the proposed optimizing cost function QCF can achieve the desired intensity precisely, whereas η and PBR cost functions overshoot the intensity limit at the focus spot.

## 4 Discussion and Conclusions

In this study, we present a QCF, which is formulated from the $\ell_{2}$-norm principles. The objective of the QCF formulation is to develop and establish a simple pixel-to-pixel level correlation with both the contrast and position of the pixels. This correlation-based QCF helps to achieve the contrast in a controlled manner and preserves the shape of the focus spot. Comprehensive experiments and simulations have been conducted to evaluate the performance of the proposed QCF in comparison to commonly used optimization cost functions, such as intensity (η) and PBR.

The findings (Figs. 2–12) indicate that the QCF outperforms other cost functions in effectively managing background intensity suppression and modulating the intensity gain of the focused spots in a controlled manner. It has been observed that the η and PBR cost function overshoot the contrast limit when considering a specific focus-spot intensity. By contrast, the proposed QCF method demonstrates enhanced efficacy in regulating both the pre-decided contrast gain and uniformity, thus enabling precise control over the desired contrast set value across the focus-spot region, surpassing the performance of the commonly utilized η and PBR cost functions.

The algorithm and cost functions are mathematically independent of the wavelength of incident light. However, the wavelength of light impacts the magnitude of scattering in the medium and, thereafter, the coherence length of scattered light. Eventually, the wavelength and scattering together impact the quality of the focus spot.

Overall, QCF emerges as the most simple and appropriate approach for achieving intensity modulation precisely at the focus spot. This method effectively minimizes background fluctuations and ensures uniform light distribution across the pixels of the focus spot. The methodology proposed in this research holds significant promise for diverse applications across multiple disciplines, such as laser materials processing, next-generation deep-tissue optical microscopy development, photolithography, photothermal treatments, dosimetry, and precise manipulation of photon energy in bio-incubation systems.

## Supplementary Material



## Data Availability

The original contributions presented in the study are included in the article and the Supplementary Material; further inquiries can be directed to the corresponding author.
