# Supplementary material for: Spatial correlation-based quadratic cost function for wavefront shaping through scattering media
Source: J Biomed Opt. 2024 Nov 20;29(11):115002. doi: 10.1117/1.JBO.29.11.115002 (PMC11576577; doi:10.1117/1.JBO.29.11.115002)
Supplement: Supplementary file 1 [file JBO_029_115002_SD001.pdf]

Supplemental material: Spatial correlation based quadratic cost function for wavefront shaping through scattering media

Amit Kumar, Ayush Sharma, S.K. Biswas*
Bio-NanoPhotonics Lab, Department of Physical Sciences, Indian Institute of Science Education and Research Mohali, Knowledge City, Sector 81, SAS Nagar, Manauli, PO 140306, India.

S1 Reference images

The reference images utilized for the experiments are shown in Fig. S1, where Fig. S1(a) shows the reference image for the focus-spot with 50 grayscale, Fig. S1(b) shows reference image for the focus-spot with 100 grayscale, Fig. S1(c) shows for the focus-spot with 150 grayscale, and Fig. S1(d) shows for the dual focus-spots with 50 and 100 grayscale.

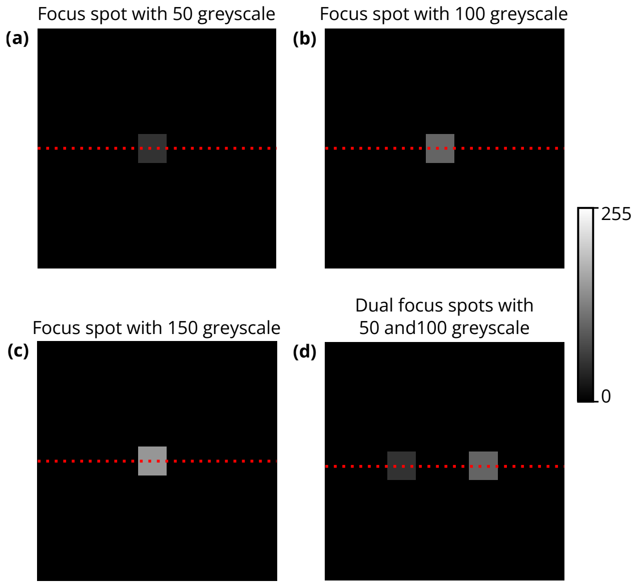

Fig S1 Reference images for single and dual focus-spots: Figure shows reference images for **(a)** focus-spot with 50 grayscale, **(b)** focus-spot with 100 grayscale, **(c)** focus-spot with 150 grayscale, and **(d)** dual focus-spots with 50 and 100 grayscale. The dashed red line indicates the horizontal cross-section used for the intensity line-plot calculation, with a vertical line-width of 6 pixels.

S2 Experimental results

S2.1 Experimental results for the GG diffuser

The experiments have also been performed for the 50 grayscale and 150 grayscale focus spots. Figs. S2 and S3 show the mean intensity analysis, final images, and lineplot analysis, respectively, for the focus-spot formation of 50 grayscale through the 220 grit GG diffuser. It is clearly evident that QCF achieves the required intensity precisely, while η and PBR surpass the required intensity and have no control over the particular intensity requirement. Similar results are also shown in Figs. S4 and S5 for the focus-spot formation of 150 grayscale through 220 grit GG diffuser.

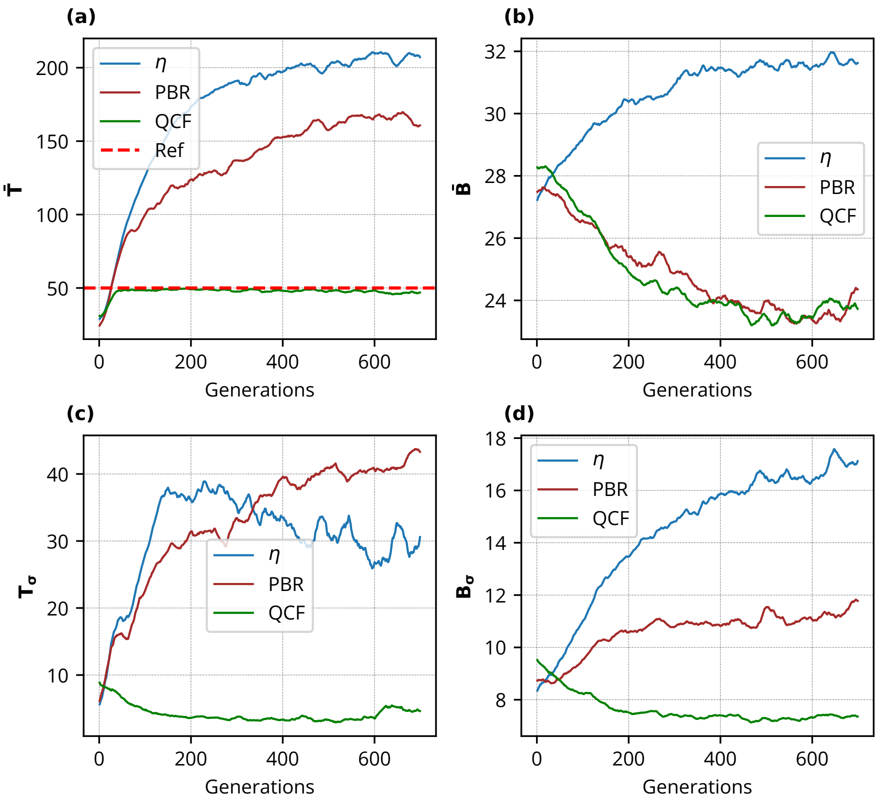

Fig S2 Experimental results for the focus-spot consisting 50 grayscale through 220 grit GG diffuser: Intensity analysis for the focus-spot consisting 50 grayscale. Where, **(a)** shows mean focus-spot intensity ($\bar{\mathrm{T}}$), **(b)** shows background intensity ($\bar{\mathrm{T}}$), **(c)** shows standard deviation of focus-spot intensity (T_σ), and **(d)** shows standard deviation of background intensity (B_σ), over generations.

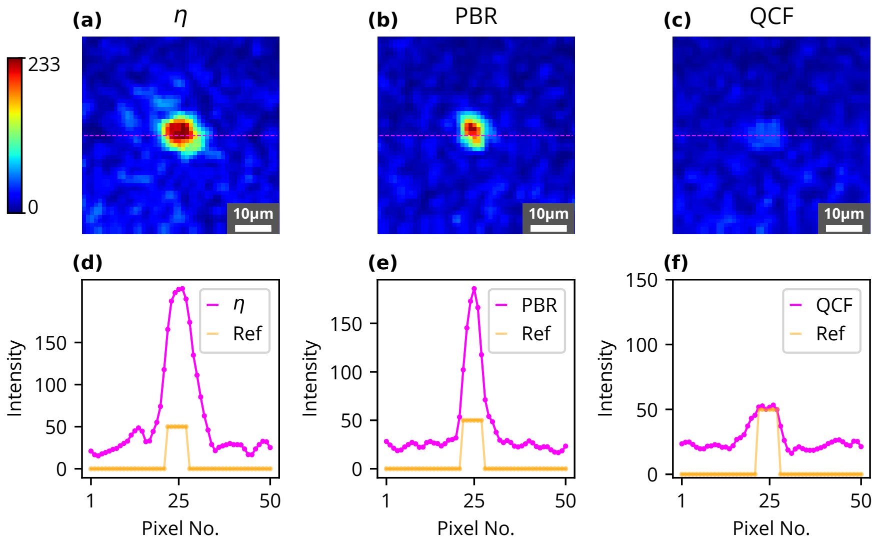

Fig S3 Experimental results for the focus-spot consisting 50 grayscale through 220 grit GG diffuser: Where, final images are shown for the focus-spot consisting 50 grayscale using **(a)** η, **(b)** PBR and **(c)** QCF. The intensity lineplots comparison for the reference image and the reconstructed image are also shown for **(d)** η, **(e)** PBR and **(f)** QCF.

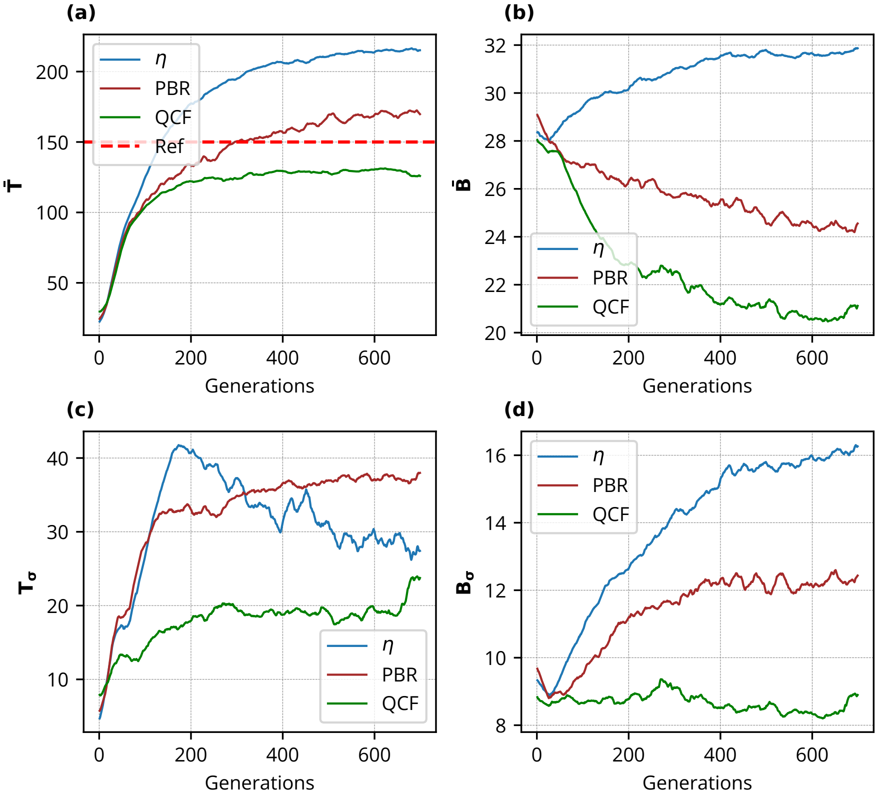

Fig S4 Experimental results for the focus-spot consisting 150 grayscale through 220 grit GG diffuser: Intensity analysis for the focus-spot consisting 150 grayscale. Where, **(a)** shows mean focus-spot intensity ($\bar{\mathrm{T}}$), **(b)** shows background intensity ($\bar{\mathrm{T}}$), **(c)** shows standard deviation of focus-spot intensity (T_σ), and **(d)** shows standard deviation of background intensity (B_σ), over generations.

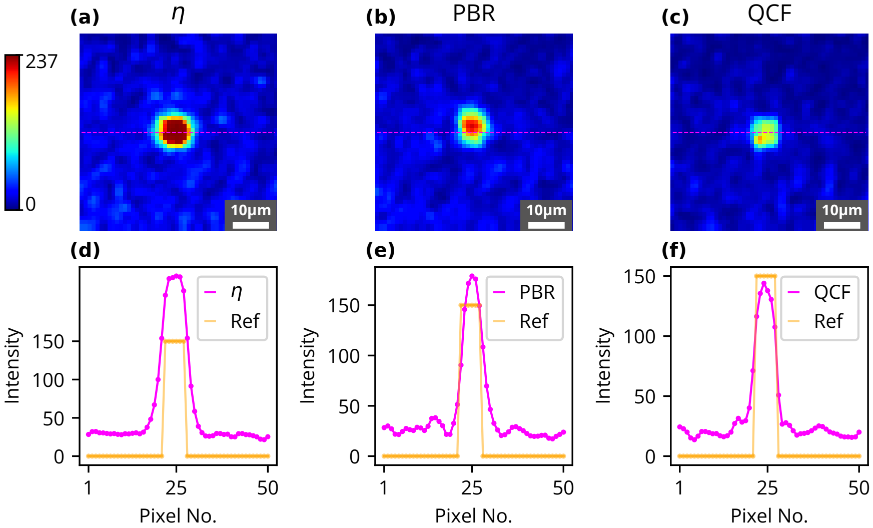

Fig S5 Experimental results for the focus-spot consisting 150 grayscale through 220 grit GG diffuser: Where, final images are shown for the focus-spot consisting 150 grayscale using **(a)** η, **(b)** PBR and **(c)** QCF. The intensity lineplots comparison for the reference image and the final reconstructed image are also shown for **(d)** η, **(e)** PBR and **(f)** QCF.

S2.2 *Grayscale scan*

Our primary goal of this article is to establish pixel-to-pixel intensity and position correlations that enable controlled contrast optimization while maintaining uniformity across the focus-spot and effectively suppressing the background intensity. We have scanned the entire grayscale starting from 10 to 255 grayscale. The details are also incorporated in Fig. S6. Our results show that with the increasing grayscale level, the efficiency of the recovered mean intensity grayscale ($\bar{I}_O$) decreases for the QCF cost function. On the other hand, η and PBR cost functions also could not obtain the mean-target intensity ($\bar{I}_O$) of 255. The η cost function achieves a mean target intensity of 215.86 averaged over 13 experimental runs, with a standard deviation of 9.46. The PBR cost function achieves a mean target intensity of 172.89 averaged over 13 experimental runs, with a standard deviation of 16.69. However, the proposed QCF method advances the photon in the focus-spot in a controlled manner, whereas the η and PBR methods fail.

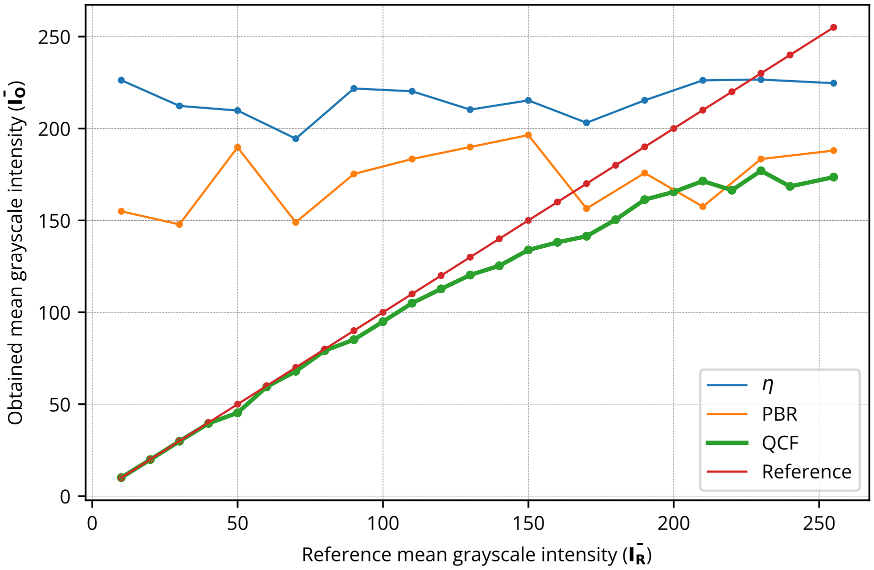

Fig S6 Reference vs. obtained mean grayscale intensity scan. Fig. shows the grayscale scan lineplot for η, PBR and QCF cost functions from grayscale values 10 to 255. Where the scan for η and PBR shows no correlation in the reference mean grayscale intensity ($\bar{I}_R$) and obtained mean grayscale intensity ($\bar{I}_O$) because there is no mathematical correlation between the provided grayscale value and the η and PBR cost functions. On the other hand, QCF shows a near-linear relationship between the required grayscale and the obtained grayscale up to the value of 150 grayscale. Thereafter, QCF starts saturating and obtains lower than required grayscale values.

S2.3 *Experimental results for chicken tissue*

The experiments have also been performed for a 50 grayscale focus-spot. Figs. S7 and S8 show the mean intensity analysis, final images, and lineplot analysis, respectively, for the focus-spot formation of 50 grayscale through the chicken tissue of 630 μm thickness. These results also clearly indicate that QCF achieves the required intensity precisely, while η and PBR surpass the required intensity and have no control over the particular intensity requirement.

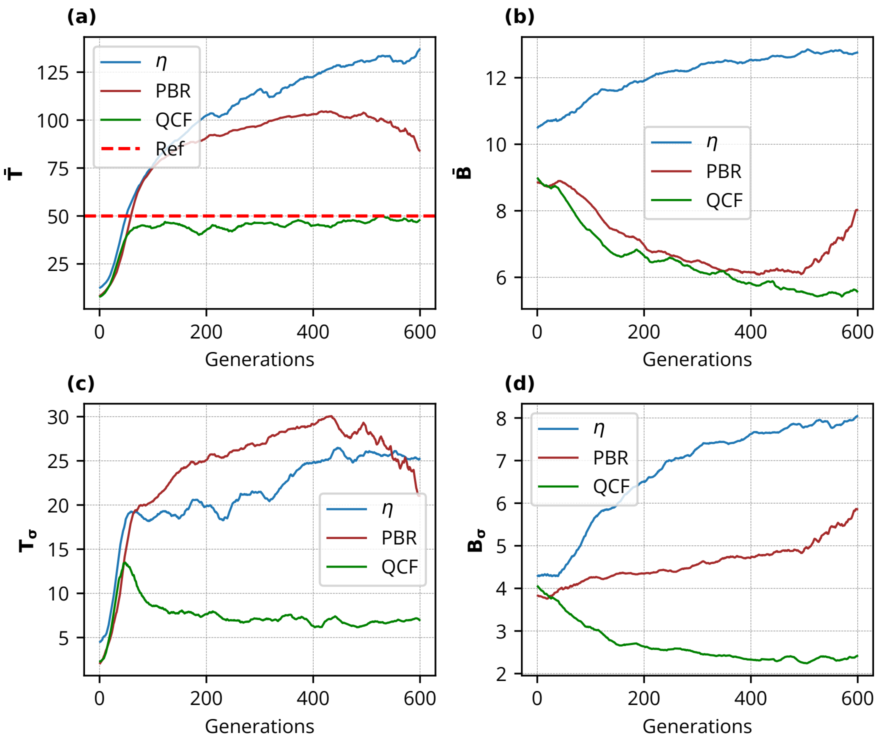

Fig S7 Experimental results for the focus-spot consisting 50 grayscale through chicken tissue of 630 µm thickness: Intensity analysis for the focus-spot consisting 50 grayscale. Where, **(a)** shows mean focus-spot intensity ($\bar{\mathrm{T}}$), **(b)** shows background intensity ($\bar{\mathrm{T}}$), **(c)** shows standard deviation of focus-spot intensity (T_σ), and **(d)** shows standard deviation of background intensity (B_σ), over generations.

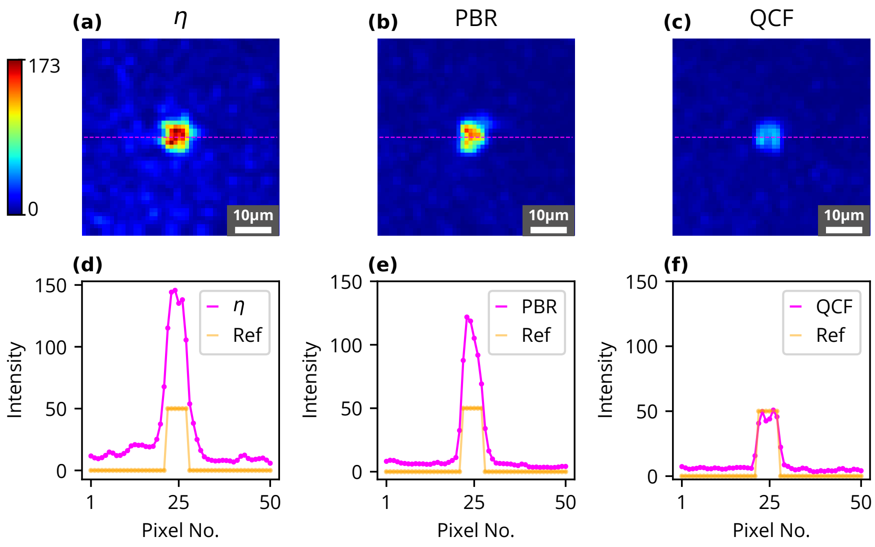

Fig S8 Experimental results for the focus-spot consisting 50 grayscale through chicken tissue of 630 µm thickness: Where, final images are shown for the focus-spot consisting 50 grayscale using **(a)** η, **(b)** PBR and **(c)** QCF. The intensity lineplots comparison for the reference image and the final obtained image are also shown for **(d)** η, **(e)** PBR and **(f)** QCF.

S3 Preparation of chicken tissue samples for the experiment

The experiment was conducted without the use of live animals. A portion of fresh, skinless chicken (age = 10 weeks, measured density = $0.92\,\mathrm{g/cm^3}$) was procured from the local market. To make slicing easier, the chicken thigh was frozen for four hours at a steady $-14\,^{\circ}\mathrm{C}$ temperature. The chicken tissue was divided into several slices using a sterile surgical scalpel. The measured thickness of the sliced chicken tissue for the experiment has been found to be 630 μm. The sliced chicken muscle was sandwiched between two microscope glass coverslips. To keep the sample from drying out and to preserve it, a drop of glycerol was added. Fig. S9 shows the photograph of the sliced chicken tissue.

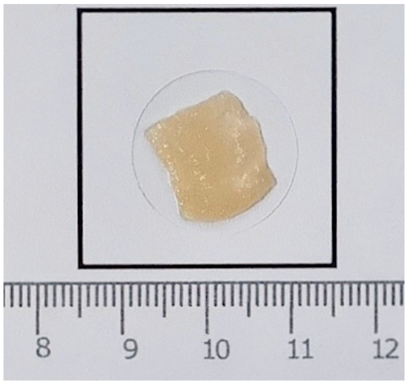

Fig S9 Image of chicken tissue slice of 630 μm thickness prepared for the experiment.

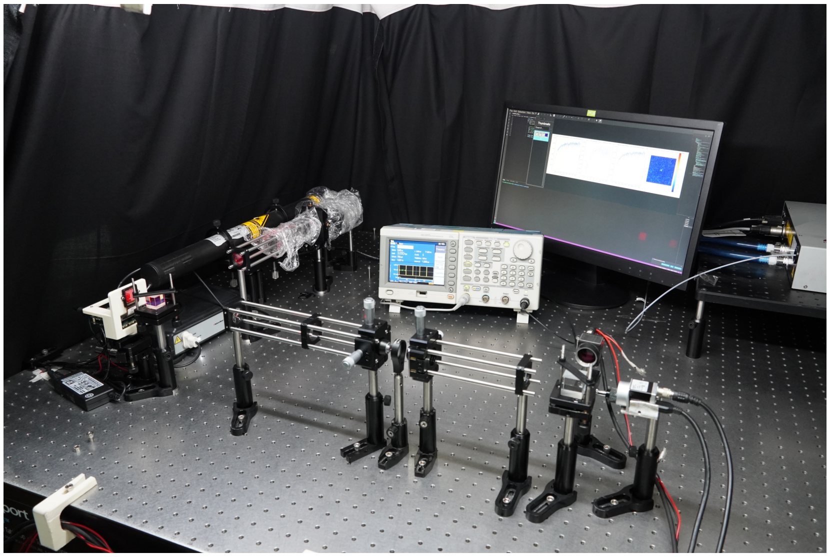

Fig S10 The photograph depicts the experimental setup featuring various optical components: Light emitted from the He-Ne laser (633 nm) is spatially filtered and directed to the SLM, where it is modulated. The modulated light is then reflected through a 4F setup and projected onto the scattering media using an objective lens. A second objective lens is positioned behind the scattering media to image the plane onto a camera placed behind the second objective and a set of optical elements. A function generator is employed to synchronize the output signal from the FLC-SLM and trigger the two cameras.
